# *Bifidobacterium* in the gut microbiota confer resilience to chronic social defeat stress in mice

**DOI:** 10.1038/srep45942

**Published:** 2017-04-03

**Authors:** Chun Yang, Yuko Fujita, Qian Ren, Min Ma, Chao Dong, Kenji Hashimoto

**Affiliations:** 1 Division of Clinical Neuroscience, Chiba University Center for Forensic Mental Health, Chiba 260-8670, Japan

## Abstract

Accumulating evidence suggests that abnormalities in the composition of the gut microbiota may play a role in the pathogenesis of depression. Although approximately 30% mice are resilient to chronic social defeat stress (CSDS), the role of gut microbiota in this stress resilience is unknown. In this study, male C57BL/6 mice were exposed to a different CD1 aggressor mouse for 10 min on 10 consecutive days. A social interaction test was applied to distinguish between resilient and susceptible mice. Using 16S rRNA analysis, we examined the composition of gut microbiota in feces from control, resilient, and susceptible mice. The marked appearance of *Bifidobacterium* was detected in the resilient mice, whereas in the control and susceptible mice, *Bifidobacterium* were below the detection limit. Oral intake of *Bifidobacterium* significantly increased the number of resilient mice after CSDS compared with vehicle-treated mice. These findings suggest that *Bifidobacterium* may confer resilience to CSDS. Therefore, supplementation of *Bifidobacterium* may prevent the onset of depression from stress in humans. In addition, supplementation of *Bifidobacterium* may prevent or minimize relapse from remission induced by inflammation and/or stress in depressed patients.

Depression is a severe and chronic psychiatric disease affecting 350 million individuals worldwide. Indeed, approximately 1 million individuals commit suicide every year^1^. Humans display wide physiological variabilities in their response to stressors after exposure to psychological stress^2^. Several lines of evidence show that resilience is mediated by adaptive changes in several neural circuits, including numerous neurotransmitters and molecular pathways^3^,^4^,^5^. However, the precise underlying mechanisms of stress resilience in psychiatric disorders, such as depression, remain obscure.

The gut-microbiota-brain axis consists of bidirectional communication between the gut and brain^6^,^7^. Accumulating evidence suggests that the brain acts on gastrointestinal and immune functions that have inherent assistance in shaping the gut’s microbial composition^8^,^9^ and that gut microbes could affect host brain functions by producing and secreting substances consisting of neurotransmitters and metabolites^10^,^11^,^12^. Recent advancements in the alleviation of psychiatric diseases by optimizing the composition of the gut microbiota have attracted attention^13^,^14^. Dysbiosis in the gut may be implicated in the development or exacerbation of depression^15^. Antidepressants have pharmacological properties that exert beneficial effects by improving gut microbiota^16^,^17^,^18^.

We previously reported that neurological processes, including, glutamatergic and γ-aminobutyric acid (GABA) ergic neurotransmissions, brain-derived neurotrophic factor, dendritic spine density, peripheral interleukin-6 (IL-6), Keap1-Nrf2 system, and soluble epoxide hydrolase, may confer stress resilience in learned helplessness and chronic social defeat stress (CSDS) models^19^,^20^,^21^,^22^,^23^,^24^. However, there are no reports showing the role of gut microbiota in stress resilience.

Therefore, the present study was undertaken to examine whether the composition of gut microbiota in the feces of resilient and susceptible mice after CSDS is altered. The gram-positive bacteria *Bifidobacterium* were detected in resilient mice, but not in control or susceptible mice. *Bifidobacterium* are non-motile bacteria^25^ that have been well established as probiotic and ubiquitous inhabitants of the gastrointestinal tract, vagina, and mouth of humans^26^. Preclinical and clinical studies show that *Bifidobacterium* may have therapeutic benefits for mood disorders^27^,^28^,^29^. Therefore, we examined whether oral intake of *Bifidobacterium* could elicit a beneficial effect in the facilitation of stress resilience.

## Results

### Effects of CSDS on the duration time spent in the interaction area

**Figure 1:**
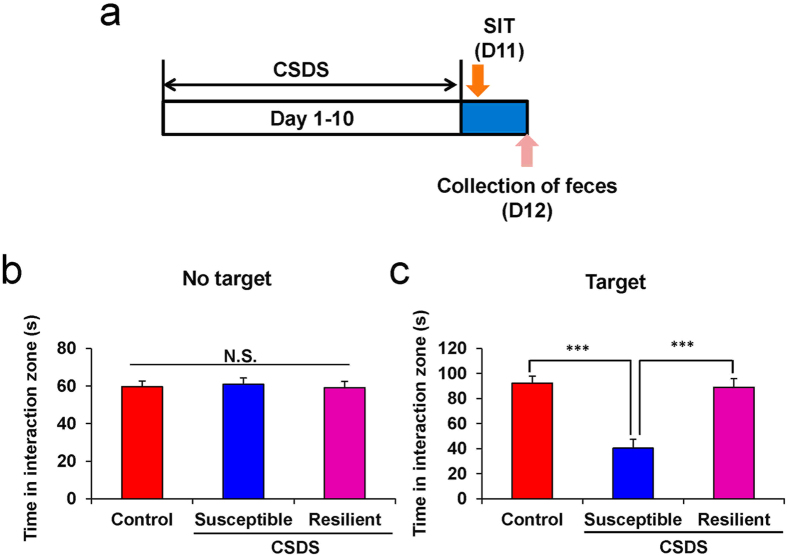
The duration the mice spent in the interaction zone of social interaction test. (**a**) The schedule of social defeat stress model (CSDS), social interaction test (SIT) and collection of feces. SIT was performed on day 11. Feces from all mice were collected on day 12. Control (n = 8), susceptible (n = 8), and resilient (n = 6) mice were used for subsequent analysis of composition of gut microbiota. (**b**) Duration of mice in the interaction zone without presence of aggressive CD1 mice. (**c**) Duration of mice in the interaction zone in the presence of aggressive CD1 mice. Data are shown as mean ± S.E.M. ***P < 0.001 compared to susceptible group of CSDS group. CSDS: chronic social defeat stress.

CSDS is an animal model of depression using social conflicts between members of the same species to generate emotional and psychological stress^19^,^21^. Here we successfully constructed the CSDS model according to our previous studies^19^,^21^. One day after CSDS, we divided susceptible and resilient mice by evaluating the time each mouse spent in the interaction area (Fig. 1a). The duration the mice spent in the interaction area in the absence of CD1 mice showed no statistical change [*F*_(2,19)_ = 0.079, *P* < 0.925] (Fig. 1b and Table S1). However, in the 2.5 min of the latter period of the social interaction test, susceptible mice significantly decreased their interaction time compared with control or resilient mice [*F*_(2,19)_ = 21.063, *P* < 0.001] (Fig. 1c and Table S1).

### Analysis of fecal bacteria

**Figure 2:**
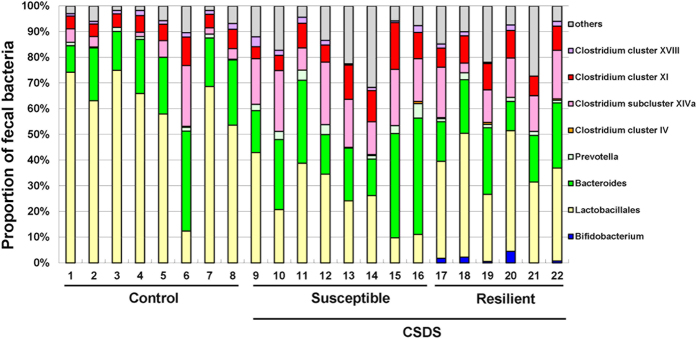
Analysis of fecal bacterium composition. Terminal Restriction Fragment Length Polymorphism (T-RFLP) was applied to analyze the fecal bacterium composition in control (n = 8), susceptible (n = 8) and resilient (n = 6) groups. CSDS: chronic social defeat stress.

**Figure 3:**
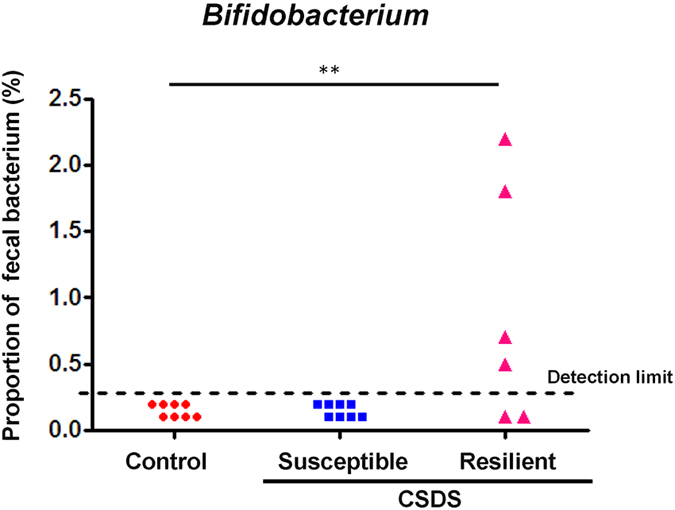
Fecal levels of *Bifidobacterium* in control, susceptible and resilient mice. Data are shown as the dot plot of each mouse (control group: n = 8, susceptible group: n = 8, resilient group: n = 6). **P < 0.01 compared to control group (Fisher’s exact test). CSDS: chronic social defeat stress.

As described in the methods, susceptible mice (<1) and resilient mice (>1) were screened based on the interaction ratio of the social interaction test. Susceptible mice showed quite a distinct profile of fecal bacteria compared with control and resilient mice (Fig. 2). The emergence of fecal *Bifidobacterium* was observed in the resilient mice, but not in control or susceptible mice. The Fisher’s exact test showed that the resilient mice had a significant increase in *Bifidobacterium* compared with control or susceptible mice (*P* < 0.01) (Fig. 3).

### Effect of oral intake of *Bifidobacterium* on social interaction and sucrose preference tests

**Figure 4:**
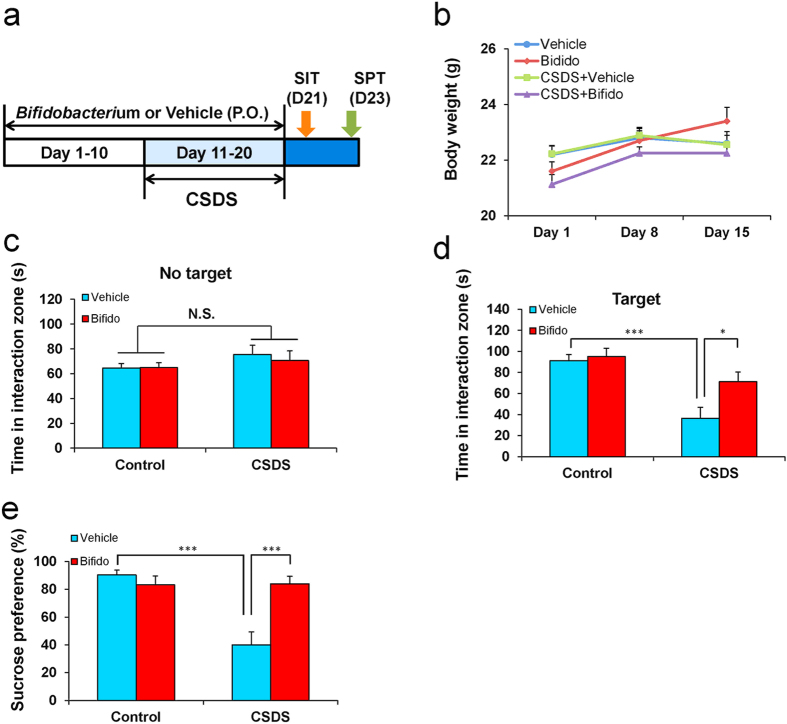
Effects of oral intake of *Bifidobacterium* on depression-like phenotype after CSDS. (**a**) The schedule of social defeat stress model (CSDS), social interaction test (SIT), sucrose preference test (SPT) and collection of feces. *Bifidobacterium* (10 mg/kg/day for 20 days) or vehicle (10 ml/kg/day for 20 days) were given orally from day 1 to day 20. CSDS was performed from day 11 to day 20. SIT and SPT were performed on day 21 and day 23, respectively. (**b**) Time course of body weight of mice. (**c**) Duration of mice in the interaction zone without presence of aggressive CD1 mice. (**d**) Duration of mice in the interaction zone in the presence of aggressive CD1 mice. (**e**) Sucrose preference test. Data are shown as mean ± S.E.M. (n = 8–10). *P < 0.05, ***P < 0.001 compared to vehicle-treated CSDS group.

To examine the prophylactic effects of *Bifidobacterium* on susceptible mice after CSDS, *Bifidobacterium* or vehicle were injected orally into mice for 20 consecutive days (Fig. 4a). There were no changes in body weight among the four groups [time: *F*_(2,11)_ = 8.137, *P* = 0.001; treatment: *F*_(3,11)_ = 2.393, *P* = 0.073; and interaction (time × treatment): *F*_(6,11)_ = 1.027, *P* = 0.413] (Fig. 4b). In the absence of CD1 mice, the intake of *Bifidobacterium* failed to show a statistical change in the duration of time the mice spent in the interaction area among the four groups [group: *F*_(1,33)_ = 2.58, *P* = 0.118; treatment: *F*_(1,33)_ = 0.359, *P* = 0.553; interaction (group × treatment): *F*_(1,33)_ = 0.064, *P* = 0.802 ] (Fig. 4c and Table S2). However, in the presence of a CD1 mouse, two-way ANOVA showed that CSDS significantly decreased the duration of time the mice spent in the interaction area [group: *F*_(1,33)_ = 20.096, *P* < 0.118; treatment: *F*_(1,33)_ = 4.339, *P* = 0.045; interaction (group × treatment): *F*_(1,33)_ = 7.82, *P* = 0.009] (Fig. 4d and Table S2).

The sucrose preference test has widely been used for the evaluation of anhedonia, which is a core symptom of depression^30^. Intake of *Bifidobacterium* for 20 consecutive days significantly attenuated the decreased sucrose preference in mice compared with that of mice with vehicle intake [group: *F*_(1,31)_ = 15.09, *P* < 0.001; treatment: *F*_(1,31)_ = 8.667, *P* = 0.006; interaction (group × treatment): *F*_(1,31)_ = 16.491, *P* < 0.001] (Fig. 4e). These results suggest that the intake of *Bifidobacterium* may prevent the onset of depression-like phenotypes (susceptibility) in mice after CSDS.

## Discussion

The major findings of this study are as follows: First, stress resilience after CSDS may be associated with the emergence of *Bifidobacterium* in the host gut. Second, oral intake of *Bifidobacterium* significantly increased the number of resilient mice after CSDS. These findings suggest that marked increases in *Bifidobacterium* may contribute to stress resilience after CSDS and that supplementation of *Bifidobacterium* may prevent the onset of depression-like phenotypes after CSDS.

*Bifidobacterium*, a genus of gram-positive anaerobic bacteria, are ubiquitous inhabitants of the gastrointestinal tract, vagina, and mouth^25^. Furthermore, *Bifidobacterium* are one of the major genera of *Actinobacteria* that make up the colon microbiota in mammals. During early infancy, *Bifidobacterium* may make up to 95% of the fecal flora of breast-fed babies. After weaning and subsequent exposure to food-derived and environmental microorganisms, the relative abundance of *Bifidobacterium* decreases. After establishment of the adult microbiota, the numbers of *Bifidobacterium* remain relatively stable at 3–6% of all bacteria. Moreover, *Bifidobacterium* may produce short-chain fatty acids to decrease the gut pH, form biological barriers, and secrete anti-microbial compounds to attenuate harmful bacteria^31^,^32^. Postnatal stress, such as mother-child separation, caused an altered composition of microbiota and decreased *Bifidobacterium* levels in the gut of rhesus monkeys^33^. A recent study showed that depressed patients have significantly lower fecal counts of *Bifidobacterium* than healthy controls, suggesting the role of *Bifidobacterium* in the pathogenesis of depression^34^. Collectively, these findings suggest that reduced composition of *Bifidobacterium* in the gut may accelerate the onset of depression.

In this study, we did not detect *Bifidobacterium* in control or susceptible mice because of the detection limits of the assay. In contrast, Friswell *et al*.^35^ reported the detection of *Bifidobacterium* in control C57BL/6 mice. Although the reason underlying this discrepancy is unclear, the method (terminal restriction fragment length polymorphism analysis) used in this study may contribute to the difference. We demonstrated marked increases in *Bifidobacterium* levels in resilient mice after CSDS, suggesting that marked increases in *Bifidobacterium* in the gut may play a role in stress resilience. *Bifidobacterium* are known to reduce intestinal endotoxin levels and improve mucosal barrier function. However, precise mechanisms underlying an increase in gut *Bifidobacterium* in resilient mice are currently unknown and further studies are needed.

Microbiota has a wide variety of physiological actions on the host^36^. Indeed, the crosstalk between the gut and brain is predominately influenced by the gut bacteria^6^,^7^,^8^,^9^,^10^,^11^. It is well established that an imbalance in gut microbiota can cause an abnormal gut-microbiota-brain axis resulting in several neurological and psychiatric diseases^6^,^7^,^37^. Accumulating evidence suggests that dysfunction of the gut-microbiota-brain axis is of substantial relevance to mood disorders^6^,^7^,^38^. An unhealthy diet has emerged as one of the significant risk factor for dysbiosis that can induce the onset of depression^38^. Recent studies showed that gut microbiota may enhance the activity of CNS by producing and secreting neuroactive substances, such as serotonin and GABA^39^,^40^. Probiotics have antidepressant-like and anxiolytic activities in rodents^41^. The effects of probiotics are strongly related to the modulation of immune and neuroendocrine systems^41^. It is also recognized that *Bifidobacterium* are the main gut bacteria involved in the positive effects observed after prebiotic supplementation. Indeed, supplementation of *Bifidobacterium* has been associated with lower bacterial translocation and endotoxaemia, leading to a decrease in inflammatory cascade activation in several models of gut bacteria translocation^42^. Given the beneficial effects of *Bifidobacterium* to human health, it is likely that supplementation of *Bifidobacterium* may improve depressive symptoms or enhance stress resilience in humans.

It has been well recognized that imbalanced inflammatory and anti-inflammatory responses are also involved in the pathogenesis of depression and targeted by the therapeutic effects of antidepressants^43^,^44^. Lipopolysaccharides (LPS), also known as lipoglycans and endotoxins, are the major component of the outer membrane of gram-negative bacteria, which has been verified to elicit several symptoms resembling depression^45^. Antidepressants not only have pharmacological benefits on depression but also have anti-inflammatory effects^46^. Elevated hypothalamic-pituitary-adrenal axis and depression-like behaviors in germ-free rats were successfully treated with *Bifidobacterium*^47^. Thus, treatment strategies targeting an increase in probiotics have led to beneficial actions on depressive symptoms and immune responses. Taken together, it is likely that supplementation of *Bifidobacterium* would have beneficial effects on the brain function by modifying the microbiota composition of the gut.

In conclusion, the present study suggests that increases in gut *Bifidobacterium* may contribute to stress resilience after CSDS. Given the role of gut *Bifidobacterium* in stress resilience, supplementation with *Bifidobacterium* may prevent the onset of depression from stress in humans. In addition, supplementation of *Bifidobacterium* may prevent or minimize relapse from remission induced by inflammation and/or stress in depressed patients.

## Materials and Methods

### Animals

Male adult C57BL/6 mice, aged 8 weeks (body weight 20–25 g, Japan SLC, Inc., Hamamatsu, Japan) and male adult CD1 (ICR) mice, aged 13–15 weeks (body weight >40 g, Japan SLC, Inc., Hamamatsu, Japan) were used. Animals were housed under controlled temperatures and 12 hour light/dark cycles (lights on between 07:00–19:00 h), with ad libitum food (CE-2; CLEA Japan, Inc., Tokyo, Japan) and water. This study was carried out in strict accordance with the recommendations in the Guide for the Care and Use of Laboratory Animals of the National Institutes of Health. The study was approved by the Chiba University Institutional Animal Care and Use Committee.

### Materials

*Bifidobacterium* (LAC-B Granular Powder, Kowa Pharmaceutical, Ltd, Tokyo, Japan) was used in this study. The dose (10 mg/kg/day for 20 days) of *Bifidobacterium* or vehicle (water, 10 ml/kg/day for 20 days) was given into C57BL/6 mice (Fig. 4a).

### Social defeat stress model

The procedure of social defeat stress was performed as previously reported^19^,^20^,^21^. Every day the C57BL/6 mice were exposed to a different CD1 aggressor mouse for 10 min, total for 10 days. When the social defeat session ended, the resident CD1 mouse and the intruder mouse were housed in one half of the cage separated by a perforated Plexiglas divider to allow visual, olfactory, and auditory contact for the remainder of the 24-h period. At 24 h after the last session, all mice were housed individually. On day 11, a social avoidance test was performed to identify subgroups of mice that were susceptible and unsusceptible to social defeat stress. This was accomplished by placing mice in an interaction test box (42 × 42 cm) with an empty wire-mesh cage (10 × 4.5 cm) located at one end. The movement of the mice was tracked for 2.5 min, followed by 2.5 min in the presence of an unfamiliar aggressor confined in the wire-mesh cage. The duration of the subject’s presence in the “interaction zone” (defined as the 8-cm-wide area surrounding the wiremesh cage) was recorded by a stopwatch. The interaction ratio was calculated as time spent in an interaction zone with an aggressor/time spent in an interaction zone without an aggressor. An interaction ratio of 1 was set as the cutoff: mice with scores <1 were defined as “susceptible” to social defeat stress and those with scores ≥1 were defined as “unsusceptible”.

### Sucrose preference test (SPT)

Mice were exposed to water and 1% sucrose solution for 48 h, followed by 4 hours of water and food deprivation and a 1 hour exposure to two identical bottles, one is water, and another is 1% sucrose solution. The bottles containing water and sucrose were weighed before and at the end of this period and the sucrose preference was determined.

### Fecal bacteria analysis

Fecal samples were collected after behavioral tests. The samples were stored in a refrigerator with a temperature of −80 °C. The analysis of fecal bacteria was performed at TechnoSuruga Laboratory Co, Ltd. (Shizuoka, Japan). DNA was automatically extracted from the processed supernatant with the use of a 12GC and GC series Magtration-MagaZorb DNA Common Kit 200N (Precision System Science, Chiba, Japan). The final concentration of the extracted DNA was adjusted to 10 ng/μL. Terminal restriction fragment length polymorphism (T-RFLP) analysis was performed according to the previous study^48^. The 16S rRNA gene was amplified with the use of primer sets 516F (5′-TGCCAGCAGCCGCGGTA-3′) and 1492R (5′-GGTTACCTTGTTACGACTT-3′). The 5′-end of the forward primer 516F was labeled with 6′-carboxyfluorescein. Amplified polymerase chain reaction (PCR) products were refined with the usage of MultiScreen^®^ PCR 96 filter plates (Millipore, Tokyo, Japan). The refined products (about 3 μL) were digested for 3 h at 55 °C with 10 U of *Bsl* I restriction enzyme (New England Biolabs, Inc., Ipswich, MA, USA). The length of the separated fluorescent PCR fragment was determined with an ABI PRISM 3130xl genetic analyzer (Applied Biosystems, Tokyo, Japan), and the data were analyzed with GeneMapper^®^ software. MapMarker^®^ X-Rhodamine Labeled 50–1000 bp (BioVentures, Inc., Murfreesboro, TN, USA) was used as a size standard marker. NTSYSpc software (Exeter Software, Setauket, NY, USA) was used to perform cluster analysis. Each terminal restriction fragment (T-RF) was expressed as a percentage of the peak area of all T-RFs. Disparity in similarity among fecal samples in individual mice was calculated using a correlation matrix and was presented graphically on tree diagrams with the use of a weighted pair-group method with arithmetic mean (WPGMA) clustering^48^.

### Statistical Analysis

The data are shown as the mean ± standard error of the mean (S.E.M.). Analysis was performed by using PASW Statistics 20 (formerly SPSS statistics; SPSS, Tokyo, Japan). Comparisons between groups were performed by one-way or two-way analysis of variance (ANOVA), followed by *post-hoc* Tukey test. Fisher’s exact test was used for comparison of proportion of fecal bacteria among the groups. Data of body weight were analyzed by repeated two-way ANOVA, followed by *post-hoc* Tukey test. The *P* < 0.05 was considered statistically significant.

## Additional Information

**How to cite this article**: Yang, C. *et al*. *Bifidobacterium* in the gut microbiota confer resilience to chronic social defeat stress in mice. *Sci. Rep.* **7**, 45942; doi: 10.1038/srep45942 (2017).

**Publisher's note:** Springer Nature remains neutral with regard to jurisdictional claims in published maps and institutional affiliations.

## Supplementary Material

Supplemental Information
