# Supplementary material for: Bifidobacterium in the gut microbiota confer resilience to chronic social defeat stress in mice
Source: Sci Rep. 2017 Apr 3;7:45942. doi: 10.1038/srep45942 (PMC5377462; doi:10.1038/srep45942)
Supplement: Supplemental Information [file srep45942-s1.pdf]

Supplemental Information

Bifidobacterium in the gut microbiota confer resilience to chronic social defeat stress in mice

Chun Yang, Yuko Fujita, Qian Ren, Min Ma, Chao Dong & Kenji Hashimoto

Division of Clinical Neuroscience, Chiba University Center for Forensic Mental Health, Chiba 260-8670, Japan

Correspondence: Kenji Hashimoto, Division of Clinical Neuroscience, Chiba University Center for Forensic Mental Health, Chiba 260-8670, JAPAN.

Tel: +81-43-226-2517; Fax: +81-43-226-2561 (e-mail: hashimoto@faculty.chiba-u.jp)

Table S1. Time in the interaction zone (sec) of social interaction test

No Target				Target			
	Control	Susceptible	Resilient		Control	Susceptible	Resilient
	65	62	71		87	56	121
	52	69	43		97	62	82
	69	55	55		93	43	73
	47	61	62		65	1	89
	55	41	63		69	26	90
	60	70	61		103	38	79
	61	62			115	39	
	69	68			100	58	
Mean	59.75	61	59.17	Mean	92.37	40.37	89
SEM	2.82	3.35	3.33	SEM	5.44	7.06	6.90

The data are from Figure 1.

Table S2. Time in the interaction zone (sec) of social interaction test

No Target					Target				
	Control		CSDS			Control		CSDS	
	Vehicle	*Bifido*	Vehicle	*Bifido*		Vehicle	*Bifido*	Vehicle	*Bifido*
	70	44	59	105		73	89	57	94
	57	58	82	40		83	156	79	106
	61	54	72	91		120	111	33	67
	63	89	70	74		78	31	48	45
	69	57	31	51		112	111	3	38
	72	80	105	81		91	79	3	93
	42	59	91	52		102	77	34	81
	83	72	70	71		79	77	12	46
	64	59	100			65	93	9	
	67	56				108	128		
Mean	64.56	64.89	75.56	70.63	Mean	91.1	95.2	30.89	71.25
SEM	3.38	3.93	7.53	7.78	SEM	5.84	10.73	8.89	9.20

The data are from Figure 4.

Bifido: Bifidobacterium
